# Personality May Confound Common Measures of Mate-Choice

**DOI:** 10.1371/journal.pone.0024778

**Published:** 2011-09-14

**Authors:** Morgan David, Frank Cézilly

**Affiliations:** 1 Université de Bourgogne, Équipe Écologie Évolutive, UMR CNRS 5561 Biogéosciences, Dijon, France; 2 Groupe de Recherche en Écologie Comportementale et Animale, Département des Sciences Biologiques, Université du Québec à Montréal, Montréal, Canada; 3 Institut Universitaire de France, Paris, France; University of Arizona, United States of America

## Abstract

The measurement of female mating preferences is central to the study of the evolution of male ornaments. Although several different methods have been developed to assess sexual preference in some standardized way, the most commonly used procedure consists of recording female spatial association with different males presented simultaneously. Sexual preference is then inferred from time spent in front of each male. However, the extent to which the measurement of female mate-choice is related to exploration tendencies has not been addressed so far. In the present study we assessed the influence of variation in exploration tendencies, a trait closely associated to global personality, on the measurement of female mating preference in the zebra finch (*Taeniopygia guttata*) using the widely used four-chamber choice-apparatus. The number of movements performed within both exploration and mate-choice apparatus was consistent within and across the two contexts. In addition, personality explained variation in selectivity, preference strength and consistency. High-exploratory females showed lower selectivity, lower preference scores and displayed more consistent preference scores. Our results suggest that variation in personality may affect the measurement of female mating preference and may contribute to explain existing inconsistencies across studies.

## Introduction

Assessing female mating preferences is of central importance in the understanding of the evolution of male ornaments through inter-sexual selection [1]. Despite extensive empirical effort [2], [3], mating preference remains one of the least repeatable behavioral trait [4]. Yet, several different experimental techniques have been developed to assess female preference in a vast array of taxa [5]. Although experiments in the field can be carried out to infer female choice [6], investigations conducted on captive animals are generally considered to allow for a better control of experimental conditions such as female motivation or male density. Female preference may then be deduced from observing the formation of sexual pairs between individuals interacting freely in a controlled environment [7], or from the female's willingness to copulate with different males presented sequentially [8], [9]. However, mate choice inferred from spatial association is the most commonly used procedure [5]. This method consists of recording, continuously or at regular intervals, the time spent in front of different males simultaneously presented to the female in different small and visually isolated enclosures [10]. All three techniques enable to link male characteristics to female sexual preference, or at least to female choice (but see [11]), and to estimate the intensity of sexual selection pressures potentially acting on male traits [12]. However, studies conducted on individuals from the same species commonly highlight variation between females in their sexual preference [13], [14]. Furthermore, inherent technical limitations remain. For instance, in spatial association tests, the measurement of female preference and selectivity is inferred from individual position within the apparatus over the course of the trial. This suggests that these measures can be dependent upon female activity or locomotor behavior during tests.

Although inter-individual differences in activity and exploratory behavior are common within species (see [15] and references therein) and are often included in the characterization of personality syndromes [16], [17], their relationship with mating behavior remains poorly investigated (but see [18]). Few studies have up to now examined the extent to which personality variation may underlie variation in mate-choice [19]. More specifically, the influence of exploration tendencies on the measurement of female mating preference has not been addressed so far, and the possibility remains that preference and selectivity reflect female spatial activity within the mate-choice apparatus.

In the present study we address this issue by testing the influence of exploration tendencies on the measurement of selectivity and preference during mate-choice trials through spatial association. We used the dimorphic zebra finch, *Taeniopygia guttata*, which is a model species for studies of sexual selection and personality [7], [17]. We first assessed exploration tendencies of females in a standard test cage with which birds were unfamiliar [20]–[22]. Female choice was then tested in a four-chamber apparatus, a device regularly used to assess female sexual preference in zebra finches [10], [23], and more generally, in vertebrates [24], [25].

## Materials and Methods

### Study subjects

Twenty-eight females and 42 males were purchased from a reliable supplier (Girardot, 21 Tichey, France) and, to avoid any stress due to isolation in this gregarious species, were maintained in a single room where all manipulations were performed. Only individuals with wild-type phenotypes were used in this experiment. All individuals were sexually experienced (i.e. underwent mate-choice trials and staged encounters with males during a previous study), to avoid the problem that females are less selective or less responsive when courted for the first time in their life [26], [27]. Birds were kept in unisex groups of two to three individuals in home cages (65×33×35 cm) containing four perches and four feeders. Room temperature was maintained at 22±2°C and the photoperiod was 13:11h light:dark (0730–2030 hours), with a 30-minute period of increasing light intensity in the morning and decreasing light intensity in the evening to imitate dawn and dusk. Each individual was identified by an orange plastic numbered ring (A.C. Hughes, Hampton Hill, U.K.; size XF), a neutral colour regarding male attractiveness [28]. Birds were provided with millet seeds, cuttlebones and water ad libitum. At the end of the experiment, birds were kept in groups of two to be used in subsequent studies. According to the “Decree No. 87-848 of October 19, 1987 relatif aux expériences pratiquées sur les animaux vertebras” on experiments involving vertebrate animals, no specific ethical approval is necessary for this type of study as it is a non-invasive observational study. Treatment of animals complied with this law and with the Université de Bourgogne's requirements concerning animal ethics.

### Assessment of exploration tendencies

Exploration tests were inspired by the classic procedure used in bird personality studies [17], [20]–[22]. Female exploration tendencies were assessed in a large, unfamiliar cage (140×140×70 cm) with opaque walls, a clear Plexiglas ceiling, and comprising five artificial trees each with four small branches. Neither food nor water was provided to prevent the focal individual to engage in any foraging activity. Before trials, birds were food-deprived for 1 h in a half-home cage, before being introduced in a black box placed against a small sliding door on one side of the apparatus. The experimenters then gently opened the door with a pulley system from outside the room, causing the focal bird to enter the apparatus. Individual behavior was then recorded during 30 minutes with a video-camera (JVC Everio GZ-MG20) placed one metre above the apparatus. Exploration tendencies were assessed twice with a period of two to four days between trials. During the analysis, the experimenter (MD) recorded the total number of movements between trees and between branches of a single tree. Exploratory tendencies further refer to the averaged cumulative number of movements performed within the apparatus [17], [20].

### Mate choice trials

**Figure 1:**
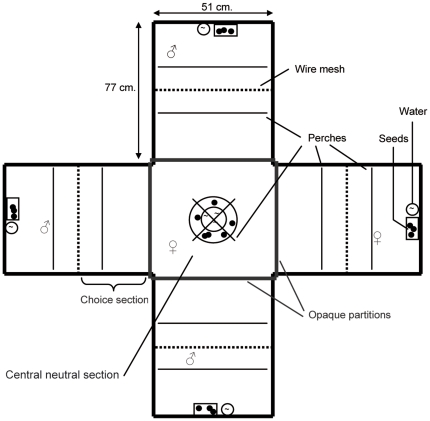
Four-chamber choice-apparatus as seen from above.

Female mate-choice was assessed in a classic four-chamber choice-apparatus (Fig. 1). The central section contained one perch and two feeders containing millet seeds and water ad libitum. One perch in each arm allowed the focal female to stand in front of each stimulus-individual. Each focal female performed three trials on three consecutive days with the same stimuli-individuals, enabling us to assess behavioral consistency. The first trial allowed the birds to acclimatize to the apparatus [10], and only the second and the third trial were video-recorded and used for data analysis. For each trial, three stimuli-males were randomly assigned to one of the four arms of the apparatus. In addition, one control-female was randomly assigned to one arm to control for focal female's sexual motivation [24], [29]. The position of stimuli-individuals was also randomized between trials. Birds were given three minutes to acclimatize to the apparatus [7]. This period proved to be sufficient for captive birds to recover from the manipulation (M. David, personal observation). Opaque partitions prevented the focal female to see the other birds during this period. Partitions were then gently removed. Female behavior was video-recorded for 30 minutes with a video-camera placed two metres above the apparatus.

The first two minutes of recording following the removal of the partitions were discarded from the analysis. Again, this period was largely sufficient for females to recover from the manipulation, i.e. resume any normal activity such as flying or calling. Moreover, stress diminution was greatly facilitated by the other birds' presence. The number of movements performed by the focal individual between each perch of the apparatus was quantified. Focal female position in the apparatus was recorded every ten seconds according to the time sampling method [30]. Each stimulus-male was used six times, i.e. three times in front of two different focal females, but within different trios of stimuli-males.

### Data analysis

Female preference was defined as the number of times observed near a given male [7]. Thus, for a given focal female, each stimulus-male was assigned a preference score ranging from zero to 180. The male obtaining the highest score during the first recorded trial was considered as the preferred mate for a given focal female. A selectivity index was defined as the standardized deviation from an egalitarian allocation of time between each stimulus-individual (termed “discrimination” in [31]), with

Selectivity index  =  (|t_1_-0.25| + |t_2_-0.25| + |t_3_-0.25| + |t_4_-0.25|)/4,

where t_i_ is the percentage of times observed near stimulus-individual i. The selectivity index varies from 0 to 0.375 with the highest value corresponding to the more selective females (i.e. spending most of their time near a single male). Number of movements within the mate-choice apparatus, preference score for the preferred male, and selectivity index were all individually averaged across the two recorded trials. Hence, “preference score for the preferred male” further refers to the average score across the two recorded trials for the male that has been preferred during the first recorded trial.

We assessed behavioral consistency (R) at the population level using the ANOVA's intra-class correlation coefficient following [32] 's method. Individual consistency in preference score was assessed by comparing the score for the preferred male during the first recorded trial and the score for the same male during the second recorded trial. We thus quantified individual consistency by extracting the absolute value of the residuals obtained from the regression of the score obtained during the second trial on that obtained during the first one. Females being more consistent were defined by low values, whereas less consistent females obtained high values.

Finally, linear regressions with “number of movements performed in the exploration apparatus” as a predictive variable were carried out to determine the influence of exploration on components of mate-choice. Analyses were all performed using JMP 5.0.1. statistical software (SAS Institute, Cary, NC, U.S.A.). Only two-tailed tests were used. Data were log or square-root transformed when necessary to reach normality. Sample size was 28 focal females for all analyses.

## Results

### Behavioral consistency

Number of movements were significantly repeatable both within the exploration apparatus (mean±95% confidence interval: 27.1±12.7; R = 0.45, *F*_27, 55_ = 2.63, *p* = 0.007), and the mate-choice apparatus (mean±95% confidence interval: 59.3±25.7; R = 0.92, *F*_27, 55_ = 24.04, *p*<0.0001). Preference scores for the preferred male and selectivity indexes were also significantly repeatable across trials (preference scores: R = 0.33, *F*_27, 55_ = 1.99, *p* = 0.037; selectivity indexes: R = 0.63, *F*_27, 55_ = 4.43, *p*<0.0001). The number of focal females preferring twice the same arm of the apparatus did not differ from the number expected by chance alone (*i.e.* 25%) (χ^2^ = 0.615, *p*>0.25), thus indicating that females were unlikely to position themselves in relation to any uncontrolled external cue.

### Personality and mate-choice

**Figure 2:**
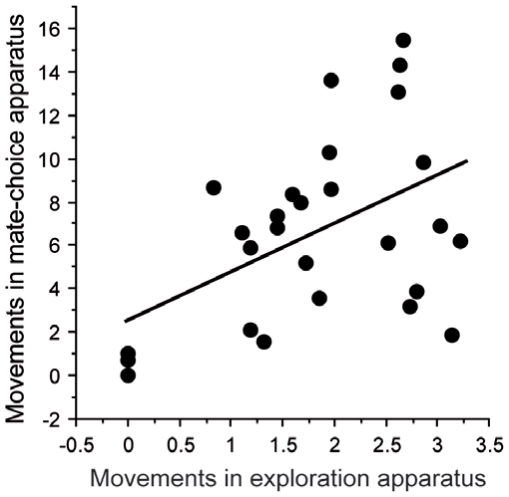
Influence of exploration tendencies on the number of movements performed during mate-choice trials. Both scales have been transformed to reach normality.

**Figure 3:**
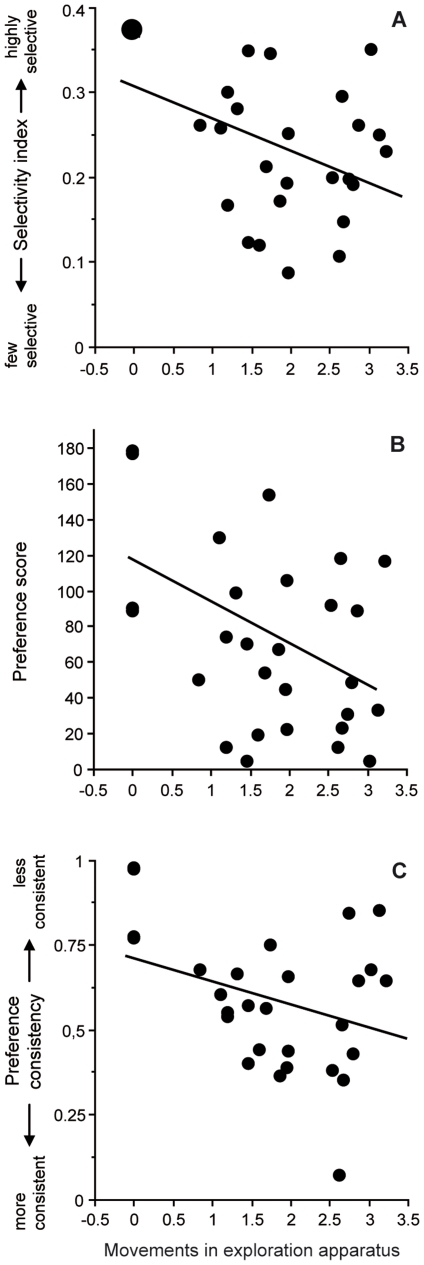
Influence of exploration tendencies on mate-choice components: selectivity index (A), average preference score for the preferred male (B), and preference consistency (C). X-axis scale has been transformed to reach normality.

The number of movements within the exploration apparatus significantly predicted the number of movements performed during mate choice trials (R^2^ = 0.25, *F*_1, 27_ = 8.76, *p* = 0.007: Fig. 2), indicating that exploration tendencies were consistent across the two contexts (R = 0.50, *F*_27, 55_ = 3.01, *p* = 0.003). The number of movements within the exploration apparatus also predicted selectivity index (R^2^ = 0.22, *F*_1, 27_ = 7.37, *p* = 0.012: Fig. 3A), preference score for the preferred male (R^2^ = 0.18, *F*_1, 27_ = 5.56, *p* = 0.026: Fig. 3B) and preference consistency (R^2^ = 0.17, *F*_1, 27_ = 5.31, *p* = 0.029: Fig. 3C). Thus, the more an individual was active within the exploration apparatus, the less it was selective during mate choice, the lower was its preference for the preferred male, but the more this preference was repeatable. Furthermore, selectivity was positively correlated to preference score for the preferred male (Pearson's r: r_27_ = 0.48, *p* = 0.010) and negatively related to preference consistency (r_27_ = −0.67, *p* = 0.0001). Finally, time spent in front of control females was not affected by exploration tendencies (R^2^<0.01, *F*_1, 27_ = 0.03, *p* = 0.85).

## Discussion

The number of movements performed within the exploration apparatus significantly predicted the number of movements performed in the mate-choice apparatus. Our results thus show that consistent variation in exploration tendencies, a trait linked to global personality in several species [17], [33], can affect the measurement of female mate preference. In addition, variation in personality influenced selectivity, preference score and preference consistency. Exploratory females allocated their time more equally between each stimulus-individual, then showed lower selectivity and lower preference scores for the preferred male, but higher preference repeatability, compared to less-exploratory females. Moreover, measures of preference and selectivity were found to be correlated to each other. This result is however not surprising, as both selectivity and preference were calculated from the number of times that focal females were observed in front of each male. Our study was not designed, however, to determine female directional preference for a particular male trait such as beak colour [23] or song quality [34], i.e. males were randomly allocated to trials, with no attempt to maximise phenotypic differences between males within a trial. In addition, the presence of a control female in one arm of the apparatus may have contributed to decrease the contrast between males, and, hence, may have affected selectivity. Yet we found no effect of focal female's personality on time spent near the control-female. On the other hand, females may actually be likely to base their preference on a combination of traits rather than on a single trait [35], such that our experimental procedure still provided the possibility that females expressed a preference for a particular male [8], [36]. Future studies should then address the influence of variation in female personality on the measurement of female preference in various contexts and with different choice apparatus.

Increasing evidence indicates that variation in personality can explain substantial inter-individual variation in behavioral performance [37]. More to the point, uncontrolled variation in personality may eventually affect the conclusions of studies in behavior and ecology. For instance, it has been shown that individuals with different personalities along the shy-bold axis have different trappability in the wild [33], [38], with obvious implications for the design of field studies and the analysis of life-history traits from capture-mark-recapture modelling [39]. Similarly, our results suggest that variation in personality may affect the measurement of female mating preference, at least in a classic spatial association setup like a four-chamber apparatus. This indicates that, in some cases, female mating behavior may hardly be distinguishable from her mere propensity to move about in an experimental apparatus. Selectivity and preference scores could simply be understood as artefacts of being more or less exploratory. Future mate-choice studies should cautiously consider the possibility that preference estimates might reflect exploration tendencies rather than an exact assessment of the propensity to mate with a given male.

Our results may then have some consequences for the design and interpretation of both experimental and field studies of female mate preference. For instance, it has been shown that differences in rearing conditions may induce variation in personality [40]. The possibility then exists that studies relying on different stocks of captive birds reared in different environments may differ in their conclusions partly because of differences in personality between groups. This could contribute to explain the recurrent inconsistencies within and between different studies [4], [13]. In zebra finches for instance, female preference for males with redder beaks, thought to be a signal of quality, is still debated [13]. Results from empirical investigations have thus provided contrasting evidence as whether redder beaks are subject to female preference or not [13]. The possibility remains that the average exploration tendencies of focal females vary between studies, which could in turn affect the conclusions. For instance, using a majority of exploratory females could lower the average preference scores measured, thus reducing the likelihood to observe a significant relationship between the presumed preferred male trait and female preference. Besides, many studies set a preference criterion under which focal females are discarded from the sample because of a presumed lack of sexual motivation [7], [41], [42]. Our results suggest that such data censoring, without any further justification, may lead to biases and non-representative estimates of female preference if females are unintentionally discriminated on the basis of their personality.

Overall, caution should be exerted when inferring female preference from spatial association measured in such experimental setups. The use of mating probability, discrete female choice or copulations might be a more reliable way to assess female mate choice [7], [26] (but see [27]). It remains yet to be shown that such indexes of female mating preference are not influenced by personality. Finally, further studies should investigate the extent to which the influence of personality on mate-choice behavior translates into different mate-sampling tactics in the wild, and its possible effects on the selection pressures applying on males [14].

## References

[1] Andersson M (1994). Sexual Selection..

[2] Andersson M, Simmons LW (2006). Sexual selection and mate choice.. Trends Ecol Evol.

[3] Jones AG, Ratterman NL (2009). Mate choice and sexual selection: what have we learned since Darwin?. PNAS.

[4] Bell AM, Hankison SJ, Laskowski KL (2009). The repeatability of behaviour: a meta-analysis.. Anim Behav.

[5] Wagner WE (1998). Measuring female mating preferences.. Anim Behav.

[6] Chaine AS, Lyon BE (2008). Adaptive plasticity in female mate choice dampens sexual selection on male ornaments in the lark bunting.. Science.

[7] Rutstein AN, Brazill-Boast J, Griffith SC (2007). Evaluating mate choice in the zebra finch.. Anim Behav.

[8] Narraway C, Hunt J, Wedell N, Hosken DJ (2010). Genotype-by-environment interactions for female preference.. J Evol Biol.

[9] Shackleton MA, Jennions MD, Hunt J (2005). Fighting success and attractiveness as predictors of male mating success in the black field cricket, *Teleogryllus commodus*: the effectiveness of no-choice tests.. Behav Ecol Sociobiol.

[10] Woodgate JL, Bennett ATD, Leitner S, Catchpole CK, Buchanan KL (2010). Developmental stress and female mate choice behaviour in the zebra finch.. Anim Behav.

[11] Heisler IL, Andersson MB, Arnold SJ, Boake CR, Borgia G (1987). The evolution of mating preferences and sexually selected traits: group report. In: Bradbury JW, Andersson MB, eds (1987) Sexual Selection: Testing the Alternatives..

[12] Hunt J, Breuker CJ, Sadowski JA, Moore AJ (2009). Male-male competition, female mate choice and their interaction: determining total sexual selection.. J Evol Biol.

[13] Collins SA, ten Cate C (1996). Does beak colour affect female preference in zebra finches?. Anim Behav.

[14] Brooks R, Endler JA (2001). Female guppies agree to differ: phenotypic and genetic variation in mate-choice behavior and the consequences for sexual selection.. Evolution.

[15] Smith BR, Blumstein DT (2008). Fitness consequences of personality: a meta-analysis.. Behav Ecol.

[16] Dingemanse NJ, Wright J, Kazem AJ, Thomas DK, Hickling R (2007). Behavioural syndromes differ predictably between 12 populations of three-spined stickleback.. J Anim Ecol.

[17] David M, Auclair Y, Cézilly F (2011). Personality predicts social dominance in female zebra finches, *Taeniopygia guttata*, in a feeding context.. Anim Behav.

[18] Schuett W, Tregenza T, Dall SRX (2010). Sexual selection and animal personality.. Biol Rev.

[19] Schuett W, Godin J-GJ, Dall SRX Do female zebra finches, *Taeniopygia guttata*, choose their mates based on their “personality”?.

[20] David M, Cézilly F, Giraldeau L-A (2011). Personality affects zebra finch feeding success in a producer-scrounger game.. Anim Behav.

[21] David M, Auclair Y, Dechaume-Moncharmont FX, Cézilly F Handling stress does not reflect personality in female zebra finches..

[22] Dingemanse NJ, Both C, Drent PJ, van Oers K, van Noordwijk AJ (2002). Repeatability and heritability of exploratory behaviour in great tits from the wild.. Anim Behav.

[23] Forstmeier W, Birkhead TR (2004). Repeatability of mate choice in the zebra finch: consistency within and between females.. Anim Behav.

[24] Pearn SM, Bennett ATD, Cuthill IC (2001). Ultraviolet vision, fluorescence and mate choice in a parrot, the budgerigar, *Melopsittacus undulatus*.. Proc Biol Sci.

[25] Hohoff C, Franzen K, Sascher N (2003). Female choice in a promiscuous wild guinea pig, the yellow-toothed cavy (*Galea musteloides*).. Behav Ecol Sociobiol.

[26] Forstmeier W (2004). Female resistance to male seduction in zebra finches.. Anim Behav.

[27] Forstmeier W (2007). Do individual females differ intrinsically in their propensity to engage in extra-pair copulations?. PLoS ONE.

[28] Burley N, Krantzberg G, Radman P (1982). Influence of colour-banding on the conspecific preferences of zebra finches.. Anim Behav.

[29] Griggio M, Biard C, Penn DJ, Hoi H (2011). Female house sparrows “count on” male genes: experimental evidence for MHC-dependent mate preference in birds.. BMC Evol Biol.

[30] Martin P, Bateson, P (1993). Measuring Behaviour: an Introductory Guide.. 2nd edn.

[31] Forstmeier W, Coltman DW, Birkhead TR (2004). Maternal effects influence the sexual behaviour of sons and daughters in the zebra finch.. Evolution.

[32] Lessells CM, Boag PT (1987). Unrepeatable repeatabilities: a common mistake.. Auk.

[33] Garamszegi LZ, Eens M, Török J (2009). Behavioural syndromes and trappability in free-living collared flycatchers, *Ficedula albicollis*.. Anim Behav.

[34] Ritschard M, Riebel K, Brumm H (2010). Female zebra finches prefer high-amplitude song.. Anim Behav.

[35] Calkins JD, Burley NT (2003). Mate choice for multiple ornaments in the California quail, *Callipepla californica*.. Anim Behav.

[36] Head ML, Hunt J, Jennions MD, Brooks R (2005). The indirect benefits of mating with attractive males outweigh the direct costs.. PLoS Biol.

[37] Réale D, Reader S, Sol D, McDougall PT, Dingemanse NJ (2007). Integrating temperament in ecology and evolutionary biology.. Biol Rev.

[38] Biro PA, Dingemanse NJ (2009). Sampling bias resulting from animal personality.. Trends Ecol Evol.

[39] Clobert J (1995). Capture-recapture and evolutionary ecology: A difficult wedding?. J Appl Stat.

[40] Carere C, Drent PJ, Koolhaas JM, Groothuis TGG (2005). Epigenetic effects on personality traits: early food provisioning and sibling competition.. Behaviour.

[41] Burley NT, Foster VS (2006). Variation in female choice of mates: condition influences selectivity.. Anim Behav.

[42] Lehtonen TK, Lindström K (2008). Repeatability of mating preferences in the sand goby.. Anim Behav.

